# CRISPR-PCDup: a novel approach for simultaneous segmental chromosomal duplication in *Saccharomyces cerevisiae*

**DOI:** 10.1186/s13568-020-0957-4

**Published:** 2020-02-03

**Authors:** Naim Hassan, Yu Sasano, Shunta Kimura, Farhana Easmin, Keisuke Ekino, Hisataka Taguchi, Satoshi Harashima

**Affiliations:** 1 Department of Applied Microbial Technology, Faculty of Biotechnology and Life Science, Sojo University, Ikeda 4-22-1, Nishi-ku, Kumamoto 860-0082 Japan; 2 Department of Biotechnology, Graduate School of Engineering, Osaka University, Yamadaoka 2-1, Suita-shi, Osaka 565-0781 Japan

**Keywords:** CRISPR/Cas9, Chromosomal Duplication, Homologous Recombination, Yeast

## Abstract

In our previous study, a novel genome engineering technology, PCR-mediated chromosome duplication (PCDup), was developed in *Saccharomyces cerevisiae* that enabled the duplication of any desired chromosomal region, resulting in a segmental aneuploid. From one round of transformation, PCDup can duplicate a single chromosomal region efficiently. However, simultaneous duplication of multiple chromosomal regions is not possible using PCDup technology, which is a serious drawback. Sequential duplication is possible, but this approach requires significantly more time and effort. Because PCDup depends upon homologous recombination, we reasoned that it might be possible to simultaneously create duplications of multiple chromosomal regions if we could increase the frequency of these events. Double-strand breaks have been shown to increase the frequency of homologous recombination around the break point. Thus, we aimed to integrate the genome editing tool CRISPR/Cas9 system, which induces double-strand breaks, with our conventional PCDup. The new method, which we named CRISPR-PCDup increased the efficiency of a single duplication by up to 30 fold. CRISPR-PCDup enabled the simultaneous duplication of long chromosomal segments (160 kb and 200 kb regions). Moreover, we were also able to increase the length of the duplicated chromosome by up to at least 400 kb, whereas conventional PCDup can duplicate up to a maximum of 300 kb. Given the enhanced efficiency of chromosomal segmental duplication and the saving in both labor and time, we propose that CRISPR-PCDup will be an invaluable technology for generating novel yeast strains with desirable traits for specific industrial applications and for investigating genome function in segmental aneuploid.

## Introduction

*Saccharomyces cerevisiae* is a model organism of immense industrial interest. It is known that many of the characteristics essential for the industrial application of *S. cerevisiae*, such as stress tolerance, are controlled by more than one gene (Swinnen et al. 2012). Consequently, genome engineering technologies are required for the rapid and effective exploitation of multiple genetic loci. Among various technologies, chromosome engineering is promising because it facilitates large scale genomic manipulation by altering chromosomes, thereby offering a powerful means of elucidating chromosome and genome function. Additionally, chromosome engineering can be used to generate useful yeast strains through the creation of a wide array of genetic diversity followed by a screening procedure to isolate the desired strains under defined culture conditions. However, a major limitation of chromosome engineering is the simultaneous manipulation of multiple chromosomal sites and regions. Previously, we developed a variety of new chromosome engineering technologies in *S. cerevisiae.* One such method, named PCR-mediated chromosome duplication (PCDup), enables the duplication of any desired chromosomal region as an independent chromosome (Natesuntorn et al. 2015). PCDup is able to duplicate chromosomal regions with lengths from 50 kb to 300 kb. Using PCDup, we discovered that segmental duplication of some chromosome regions leads to an enhanced resistant phenotype when the cells are grown under stress conditions. However, the PCDup method has limitations because duplication is restricted to a single region at each transformation step. Simultaneous duplication of two or more target regions in the genome of an organism, even in the yeast genome, has not been achieved. Time is also a major consideration when conducting genome engineering. For example, one round of duplication takes at least 11 days including confirmatory analysis and if the results is in failure, constructing stains by successive multiple chromosome duplications is both time consuming and laborious.

PCDup technology is based on the mechanism of homologous recombination. We reasoned that an improvement of homologous recombination activity might be the key to enhancing chromosome duplication efficiency. It has previously been shown that induction of double-strand breaks (DSBs) can increase recombination efficiency near the site of the DSB by as much as 4000-fold (Storici et al. 2003). Recently, RNA-guided programmable CRISPR/Cas systems have played a major role in facilitating precision genome engineering by sequence-specific introduction of double-strand breaks (DSBs) (Cong et al. 2013; Jinek et al. 2012; Sander and Joung 2014). Moreover, the CRISPR/Cas9 system has been shown to be functional in *S. cerevisiae* (DiCarlo et al. 2013). Thus, this method permits induction of site specific DSBs using an appropriate guide RNA (gRNA).

In this study, we introduced DSBs into the genome of *S. cerevisiae* using the CRISPR/Cas9 system before attempting a chromosome duplication. We show that the integration of CRISPR/Cas9 into our PCDup system, which we called CRISPR-PCDup, produces an effective genome engineering technology that enhances chromosomal duplication efficiency with a high level of fidelity and is capable of simultaneously targeting multiple chromosomal regions.

## Materials and Methods

### Strains and media

Strains used in this study are listed in Table 1. FY833 and FY834 cells containing plasmid p414-TEF1p-Cas9-CYC1t were used as a host strain (SJY415 and SJY30, respectively) for the CRISPR-PCDup experiments. *Escherichia coli* DH5α was used for plasmid construction and propagation. *E. coli* recombinant strains were grown in Luria-Bertani (LB) medium containing 100 µg/ml ampicillin. Yeast cells are grown in YPDA medium containing 1% Bacto-Yeast Extract (BD Bioscience, San Jose, CA), 2% Bacto-Peptone (Difco, Detroit, MI), 2% glucose (Wako, Tokyo, Japan), 2% agar (Wako) and 0.004% adenine sulfate (Wako) and in Synthetic Complete (SC) medium containing 0.67% Yeast Nitrogen Base without Amino Acids (Difco), 0.2% dropout mix of amino acids and nucleic acid bases and 2% glucose. SC medium lacking specific amino acids was used for the selection of transformants. For sporulation, diploid strain was cultivated in sporulation medium containing 1% potassium acetate, 0.1% bacto-yeast extract and 0.05% glucose. Agar (2% w/v) was included for solid medium.

**Table 1:** Strains and plasmids used in the CRISPR-PCDup experiments

Strain or plasmid	Description	Remarks
Strain		
FY833	*MATa ura3-52 his3-Δ200 leu2Δ1 lys2Δ202 trp1Δ63*	(Winston et al. 1995)
SJY415	Trp+ transformants of FY833 harboring plasmid p414-TEF1p-Cas9-CYC1t	This study
FY834	*MATα ura3-52 his3-Δ200 leu2Δ1 lys2Δ202 trp1Δ63*	(Winston et al. 1995)
SJY30	Trp+ transformants of FY834 harboring plasmid p414-TEF1p-Cas9-CYC1t	(Sasano et al. 2016)
Plasmid		
pUG6	Containing loxP-flanked marker gene deletion cassette: loxP-pAgTEF1-kanMX-tAgTEF1-loxP	(Güldener et al. 1996)
p3009	The *loxP-CgHIS3-loxP* module containing plasmid constructed by modifying pUG6	(Sugiyama et al. 2005)
p3121	The *CEN4* module containing plasmid constructed by modifying pUG6	(Sugiyama et al. 2005)
p3122	The *CEN4*-*loxP-CgLEU2-loxP* module containing plasmid constructed by modifying pUG6	(Sugiyama et al. 2008)
p3123	The *CEN4*-*loxP-CgHIS3-loxP* module containing plasmid constructed by modifying pUG6	(Sugiyama et al. 2008)
pSJ23	A derivative of pUG6 carrying *URA3*	(Easmin et al. 2019b)
pSJ69	loxP site-deleted p3008	(Easmin et al. 2019a)
pSJ70	loxP site deleted p3009	(Easmin et al. 2019b)
p414-TEF1p-Cas9- CYC1t	*TEF1*p-Cas9*-CYC1*t module containing YCp type plasmid	(DiCarlo et al. 2013)
p426-SNR52p-gRNA.CAN1.Y-SUP4t	SNR52p-gRNA.*CAN1* Y*-SUP4*t module containing YEp type plasmid	(DiCarlo et al. 2013)

### CRISPR-PCDup

**Fig. 1:**
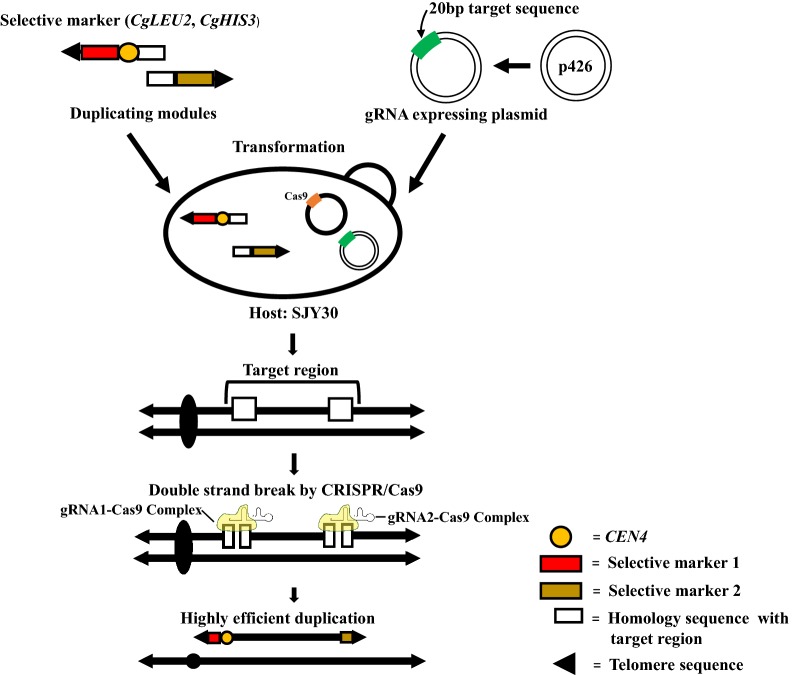
Outline of the CRISPR-PCDup method. gRNA expressing plasmid and duplicating modules are introduced into the SJY30 strain, which harbors a Cas9-expressing plasmid. In transformed cells, CRISPR/Cas9 mediated double-strand breaks (DSBs) are induced near the target site. Chromosome duplication is then facilitated by PCDup. This new technology is named CRISPR-PCDup

Details of the conventional PCDup technology for chromosome duplication has been described previously (Natesuntorn et al. 2015). Briefly, two DNA modules necessary for duplication were prepared as follows. Each DNA module has 50 bp homology sequence with the target and additionally contains either a selective marker (*CgLEU2* or *CgHIS3* or *URA3*) along with a telomere seed sequence (six copies of a 5´-CCCCAA-3´) or a centromere along with or without selective marker (*CgLEU2* or *CgHIS3*) and telomere seed sequence. p3121 (Sugiyama et al. 2005) was used as a template to add only centromere. p3122 (Sugiyama et al. 2008) was used to add centromere along with *CgLEU2.* p3123 (Sugiyama et al. 2008) was used to add centromere along with *CgHIS3* to the module. p3009 (Sugiyama et al. 2005) was used as a template to prepare the duplication module containing *CgHIS3*. *CEN4* sequence was added to one of the DNA modules so that the resulting new chromosomes possessed one centromere. Template plasmids used for targeting chromosomal regions are listed in Table 1. Primers for constructing the DNA module are listed in Additional file 1: Table S1. gRNA expression plasmids were constructed according to Sasano et al. (2016) and the software CRISPRdirect (https://crispr.dbcls.jp/) was used to select the 20 bp target sequences. Oligonucleotide primers used for construction of gRNA expression plasmids are listed in Additional file 1: Table S2. For targeting each chromosomal region, two gRNA expressing plasmids (Sasano et al. 2016) were introduced (7.5 μg each) along with the corresponding duplicating DNA modules into the transformation mixture. An outline of the CRISPR-PCDup method is shown in Fig. 1.

### Yeast and *E. coli* Transformation

*S. cerevisiae* was transformed using the lithium acetate method (Gietz and Schiestl 2007). After transformation, SC medium lacking the appropriate amino acids was used for selection of transformants having the marker gene from the duplicating module. *E. coli* was transformed according to the method described by Easmin et al. (2019a).

### Colony PCR, pulse field gel electrophoresis (PFGE) and Southern hybridization

Colony PCR was performed according to the method described by Easmin et al. (2019a). Pulse field gel electrophoresis and Southern hybridization were performed according to Sasano et al. (2016). The oligonucleotide primers used for amplifying DNA fragments for probes in Southern hybridization are shown in Additional file 1: Table S3.

### Tetrad analysis

Tetrad analysis was done according to Sugiyama et al. (2006).

## Results

### Increasing duplication efficiency by CRISPR-PCDup

We previously developed PCDup technology, which allows duplication of any desired chromosomal region of the *S. cerevisiae* genome (Natesuntorn et al. 2015). In this study, we developed CRISPR-PCDup technology, which is an integration of PCDup with CRISPR/Cas9 that facilitates simultaneous and multiple duplication of chromosome segments in *S. cerevisiae*. We reasoned that integration of the CRISPR/Cas9 system with PCDup might increase the frequency of homologous recombination, thereby enabling simultaneous duplication. Initially, we examined whether the CRISPR-PCDup method works more efficiently compared with the previous PCDup technology.

**Fig. 2:**
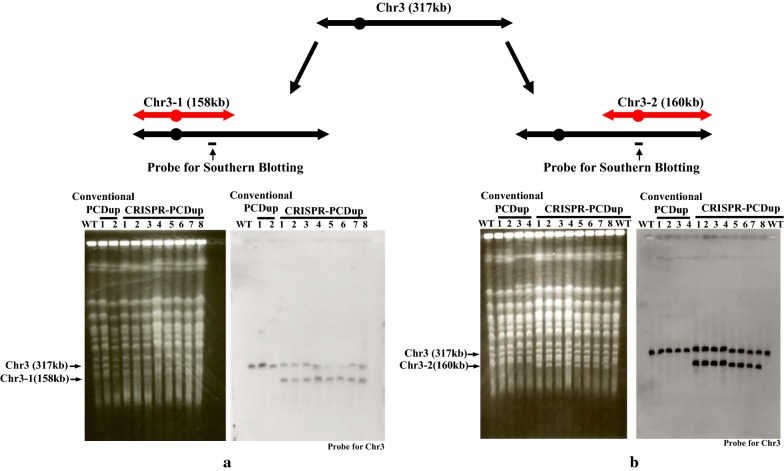
Duplication of the Chr3-1 and Chr3-2 region. The Chr3-1 and Chr3-2 regions of Chromosome 3 were chosen for the initial experiments. Both duplicating modules were prepared so as to be marked with *CgHIS3* and *CEN4*+*CgHIS3,* respectively. After transformation, two chromosomes of 158 kb (Fig. 2**a**) and 160 kb (Fig. 2**b**) were expected to be generated from Chr3-1 and Chr3-2, respectively. The left and right panel of Fig. 2**a** and Fig. 2**b** are PFGE along with the corresponding Southern blot analysis of wild type SJY30; 2 and 4 transformants selected from the conventional PCDup experiment for the Chr3-1 and for the Chr3-2 region, respectively and 8 transformants randomly selected from CRISPR-PCDup for both the Chr3-1 and Chr3-2 regions. Right panel of Fig. 2**a** and Fig. 2**b** shows the results of Southern blot analysis for detecting the 317 kb Chromosome 3 and newly duplicated 158 kb and 160 kb chromosomes, respectively

Cas9-expressing strain (SJY30) was used as host strain for chromosome segmental duplication. SJY30 showed no significant growth defect, which suggested that Cas9 expression is not toxic in this strain. We designed gRNA targeting sequences located just outside of the duplicating region and near both edges. The genomic positions chosen for duplication in this study and the gRNA targeting sequences are shown in Table 2 and Additional file 1: Table S4, respectively. Initially we attempted to produce duplication of the Chr3-1 region (1-158020) and Chr3-2 region (157543-316620) on Chromosome 3 separately (Fig. 2). SJY30 strain was transformed with gRNA expressing plasmids and two kinds of duplication modules marked with *CgHIS3* for Chr3-1 and *CEN4*+*CgHIS3* for Chr3-2. Target sequences on these gRNA-expressing plasmids were located near the edge of the Chr3-1 and Chr3-2 regions (Additional file 1: Table S4). When the CRISPR-PCDup system was employed, a total of 62 and 1316 His^+^ transformants were obtained for the Chr3-1 and Chr3-2 region, respectively (Table 3). By contrast, using 50 bp homology sequence with the target, conventional PCDup gave only 2 and 51 His^+^ transformants under the same transformation conditions for the Chr3-1 and Chr3-2 region, respectively. We chose 8 transformants at random from those obtained by CRISPR-PCDup for the Chr3-1 and Chr3-2 region and subjected them to pulse field gel electrophoresis (PFGE) and subsequent Southern blot analysis to determine whether the duplication event had occurred at the expected locus. The results of this analysis showed that all 8 transformants for duplication of the Chr3-1 and Chr3-2 regions had the expected duplicated chromosome (Fig. 2; i.e. 158 kb band in panel A and 160 kb band in panel B) in addition to intact Chromosome 3 (317 kb band). However, using conventional PCDup, 2 and 4 transformants were analyzed for the Chr3-1 and Chr3-2 region, respectively, but none had the expected duplicated chromosome (Fig. 2a, b). Based on these observations, we concluded that CRISPR-PCDup efficiently enhanced segmental duplication of a single chromosomal region.

**Table 2:** Duplication of various chromosomal regions

Duplication event	Name of target regions	Size and coordinate number of the target region	Plasmids used for preparation of the duplicating module
Single duplication	Chr3-1	Chr3 (1 - 158020) (158 kb)	p3009
	Chr3-2	Chr3 (157543 - 316620) (160 kb)	p3123
	Chr5-3	Chr5 (398496-576874) (177 kb)	pSJ70
	Chr15-L1	Chr15 (569775 - 969009) (400 kb)	p3009, p3122
	Chr15-L2	Chr15 (618914 - 969009) (350 kb)	p3009, p3122
	Chr15-L3	Chr15 (670548 - 969009) (300 kb)	p3009, p3122
	Chr15-L4	Chr15 (718509 - 969009) (250 kb)	p3009, p3122
	Chr15-L5	Chr15 (767986 - 969009) (200 kb)	p3009, p3122
Simultaneous double duplication	Chr3-2 and Chr15-L5	Chr3 (157543 - 316620) (160 kb) and Chr15 (767986 - 969009) (200 kb)	p3122 and p3009, p3121
Sequential duplication	Chr3-1	Chr3 (1 - 158020) (158 kb)	pSJ69
	Chr8-1	Chr8 (1-202241) (200 kb)	pSJ70
	Chr14-4	Chr14-4 (597394-784333)	pSJ23

**Table 3:** CRISPR-PCDup increases duplication efficiency and induces simultaneous double duplication

Duplicated regions	Method	Number of transformants (n)	Karyotype analysis (n)	Correct transformants (n)
Chr3-1 (158 kb)	CRISPR-PCDup	62	8	8 (100%)
	Conventional PCDup	2	2	0 (0%)
Chr3-2 (160 kb)	CRISPR-PCDup	1316	8	8 (100%)
	Conventional PCDup	51	4	0 (0%)
Chr3-2 (160 kb) and Chr15-L5 (200 kb)	CRISPR-PCDup	75	25	10 (40%)
	Conventional PCDup	0	–	–

Symbol “–” indicates that karyotype analysis and counting the number of correct transformants were not applicable

### Simultaneous double duplication by CRISPR-PCDup

**Fig. 3:**
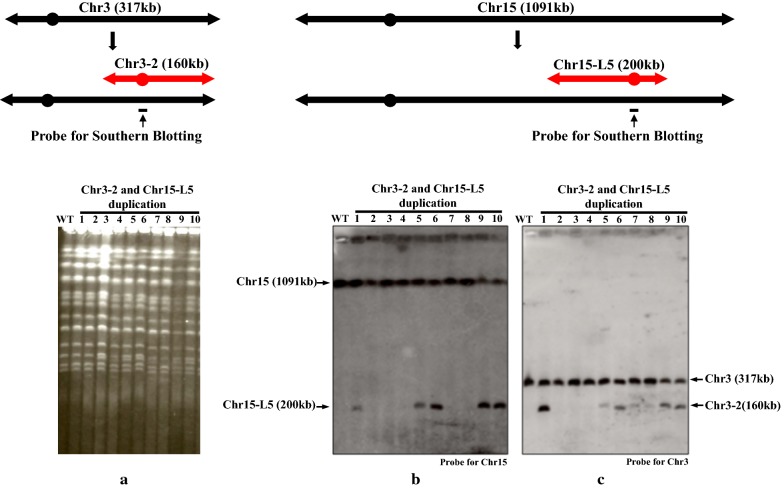
Simultaneous double duplication in the Chr3-2 and Chr15-L5 regions. The Chr3-2 region of Chr3 and the Chr15-L5 region of Chr15 were simultaneously duplicated by CRISPR-PCDup. The duplicating module of Chr3-2 was marked with *CEN4* and *CgLEU2*; C15-L5 was marked with *CEN4* and *CgHIS3*. After duplication, 2 derived chromosomes are expected to be generated; 160 kb from the C3-2 region and 200 kb from the C15-L5 region. Fig. 3**a** represents PFGE analysis of the wild-type strain, SJY30 and 10 randomly chosen transformants. Fig 3**b**,** c** shows the results of Southern blot analysis for detecting Chr15 (1091 kb) and the newly duplicated 200 kb chromosome along with Chr3 (317 kb) and the newly duplicated 160 kb chromosome, respectively

Despite numerous attempts, simultaneous duplication of two different genomic regions by conventional PCDup has never been achieved. The results in the previous section revealed that the duplication of a single chromosomal region was possible at high frequency. Next, we attempted to induce a simultaneous duplication of two genomic regions on different chromosomes, namely Chr3-2 (160 kb) and Chr15-L5 (200 kb) (Fig. 3), using our new CRISPR-PCDup approach. We obtained 75 His^+^ Leu^+^ transformants using CRISPR-PCDup whereas no transformants were obtained using conventional PCDup (Table 3). Of the 75 His^+^ Leu^+^ transformants obtained by CRISPR-PCDup, 25 were randomly selected and analyzed using PFGE and Southern blot analysis to verify whether or not the anticipated double duplication had occurred. Ten of the transformants showed double duplication as evidenced by the presence of a 200 kb and 160 kb band. Fig. 3a–c, show 10 out of 25 candidate transformants analyzed by PFGE and Southern blotting. Results shows that five out of 10 transformants had a double duplication. When we analyzed remaining 15 transformants by PFGE and Southern blotting, we found that five transformants had also undergone a double duplication while other 10 transformants had either a single duplication or no duplication event (data not shown). Therefore, we conclude that simultaneous double duplication is possible by using CRISPR-PCDup.

### Synthetic lethality is not caused by simultaneous double duplication

After successfully duplicating two chromosomal regions simultaneously using CRISPR-PCDup technology, we next attempted to duplicate other chromosomal regions in two different chromosomes, namely Chr3-1 (158 kb) (1-158020) and Chr8-1 (200 kb) (1-202241). We used DNA modules harboring the *CgLEU2* marker to duplicate the Chr3-1 region and the *CgHIS3* marker to duplicate the Chr8-1 region. Leucine- and histidine-minus (-Leu-His) medium was used to subsequently select His^+^ Leu^+^ transformants. 15 His^+^ Leu^+^ transformants were obtained and these transformants were checked by colony PCR. None of the 15 transformants showed simultaneous duplication of the Chr3-1 and Chr8-1 regions. Next, another combination comprising the Chr3-1 and Chr14-4 (184 kb) (597394-784333) regions were chosen for simultaneous duplication mediated by CRISPR-PCDup. We used DNA modules harboring the *CgLEU2* marker to duplicate the Chr3-1 region and the *URA3* marker to duplicate the Chr14-4 region. Leucine- and uracil- minus (-Leu-Ura) medium was used to select Leu^+^Ura^+^ transformants. 66 Leu^+^Ura^+^ transformants were isolated and 15 transformants subsequently checked by colony PCR. The results revealed that none of the 15 transformants showed simultaneous duplication of the Chr3-1 and Chr14-4 regions.

**Fig. 4:**
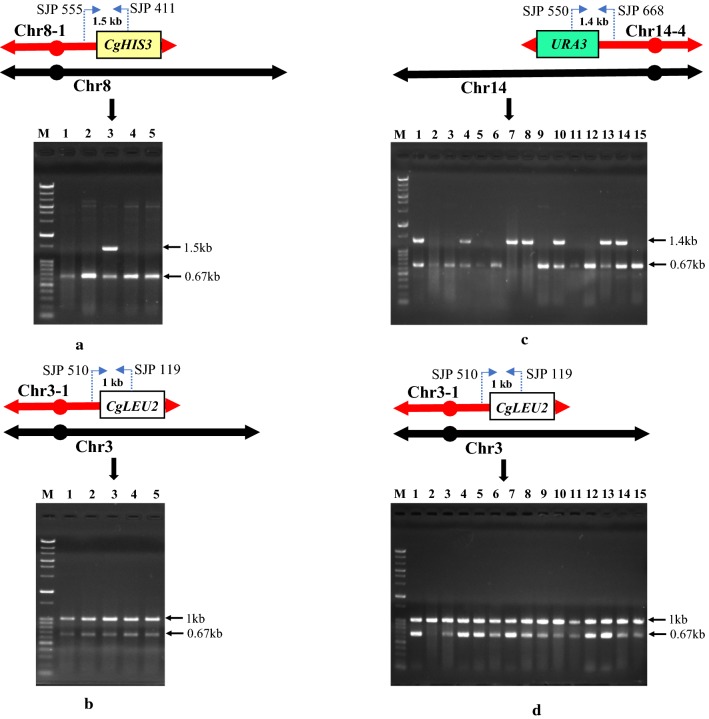
Colony PCR analysis of Chr3-1 and Chr8-1 as well as Chr3-1 and Chr14-4 sequentially duplicated transformants. In the colony PCR, each lane represents independent transformants. Fig. 4**a**,** b** represents the sequential duplication of the Chr3-1 and Chr8-1 regions, respectively. In Fig. 4**a**, primers SJP 555 and SJP 411 were used to amplify the 1.5 kb band from the duplicated Chr8-1 region and in Fig. 4b, primers SJP 510 and SJP 119 were used to amplify the 1 kb band from the duplicated Chr3-1 region. Fig. 4**c** ,** d** represents the sequential duplication of the Chr3-1 and Chr14-4 regions, respectively. In Fig. 4**c**, primers SJP 550 and SJP 668 were used to amplify the 1.4 kb band from the duplicated Chr14-4 region and in Fig. 4**d**, primers SJP 510 and SJP 119 were used to amplify the 1 kb band from the duplicated Chr3-1 region. In all PCR analysis the 0.67kb *CNE1* gene on Chromosome 1 was also amplified as an internal control by a common set of primers SJP 121 and SJP 242

Unsuccessful double duplication may be due to synthetic lethality caused by simultaneously duplicating these two sets of chromosomal regions. To investigate this possibility, we attempted to construct the double duplication in a sequential manner. Initially, the Chr3-1 region was duplicated and an attempt was made to duplicate the Chr8-1 region. In all, 155 His^+^Leu^+^ transformants were obtained and 5 transformants were arbitrarily picked for colony PCR analysis. The colony PCR revealed that of the 5 transformants, 1 gave the expected result (Fig. 4a, b). We also attempted to sequentially duplicate the Chr14-4 region in the Chr3-1 duplicated transformants. In total, 796 Leu^+^Ura^+^ transformants were isolated and 15 were arbitrarily picked for colony PCR. Of these, 7 transformants had the expected double duplication (Fig. 4c, d). Primers used for colony PCR are listed in Additional file 1: Table S5.

These findings indicated that sequential double duplication of the Chr3-1 and Chr8-1 regions as well as the Chr3-1 and Chr14-4 regions is possible. We also used an alternative approach to further confirm synthetic lethality is not caused by simultaneous double duplication. Tetrad analysis of diploids was conducted to investigate whether double duplication causes synthetic lethality or not by mating transformants harboring two single duplicated regions. For this purpose, mating type α host SJY30 was chosen and the Chr3-1 region was duplicated using the *CgLEU2* harboring DNA module. In a separate experiment, we took mating type **a** host SJY415 and duplicated the Chr5-3 (177 kb) region with the *CgHIS3* harboring DNA module (data not shown). A diploid is then constructed by mating two transformants harboring either the Chr3-1 or Chr5-3 duplicated regions. After making diploids, tetrad analysis was performed. This analysis revealed that Leu^+^His^+^ spores were viable, confirming that double duplication of these two regions is not lethal. Overall, these observations suggest that unsuccessful obtaining of double duplication is not due to synthetic lethality caused by double duplication. We will discuss possible reason for this observation in Discussion section.

### Upper size limit of duplication by CRISPR-PCDup

**Fig. 5:**
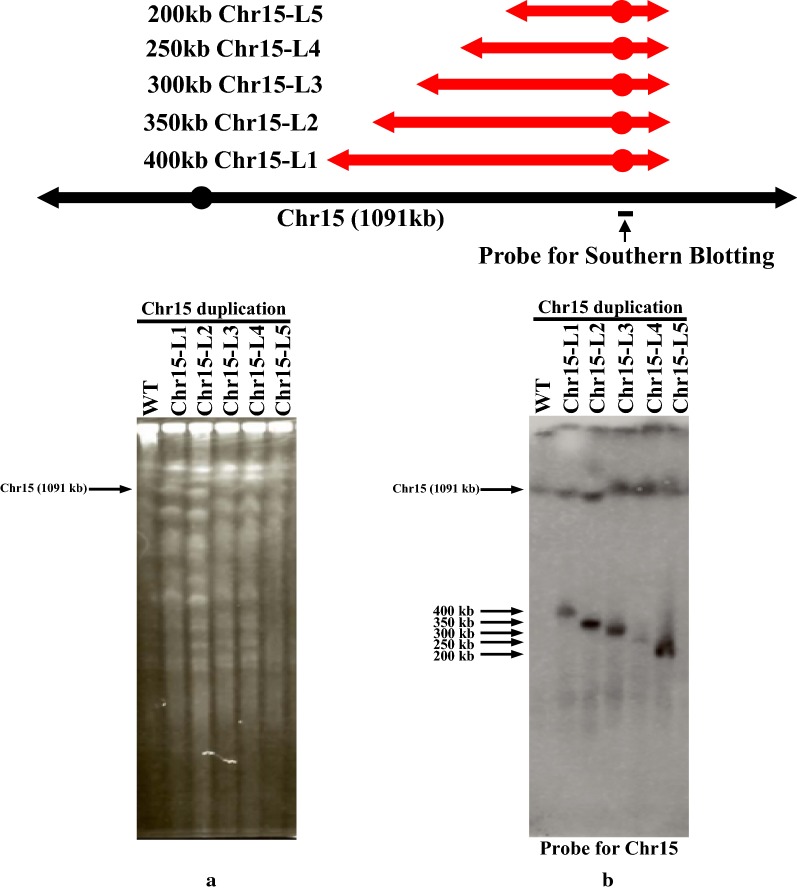
Duplication of the 200 kb to 400 kb chromosomal region in Chr15. Region Chr15-L1 to Chr15-L5 of Chr15 were selected. All the duplicating modules were marked with *CgHIS3* along with *CEN4* and *CgLEU2*. Fig. 5**a** represents PFGE analysis of wild type SJY30 and transformants obtained from the duplication experiments for the Chr15-L1, Chr15-L2, Chr15-L3, Chr15-L4 and Chr15-L5 regions by CRISPR-PCDup. Fig. 5**b** shows the results of Southern blot analysis after PFGE for the detection of the newly generated 400 kb, 350 kb, 300 kb, 250 kb and 200 kb chromosomes, respectively

We previously reported that up to 300 kb of chromosomal region could be duplicated by conventional PCDup (Natesuntorn et al. 2015). However, in this study using CRISPR-PCDup we successfully duplicated single chromosomal regions more efficiently than using the conventional PCDup procedure. Thus, we examined whether the upper size limit of the duplicated regions is increased using CRISPR-PCDup technology. For this purpose, we attempted to construct a series of segmentally duplicated chromosomes of increasing size (200 kb, 250 kb, 300 kb, 350 kb and 400 kb of Chr15), (Table 4). We found that all the regions could be duplicated (Fig. 5a, b). In the case of conventional PCDup, we did not get any transformants for the duplication of 200 kb to 400 kb. Previously, Natesuntorn et al. (2015) was able to duplicate up to 300 kb using conventional PCDup by employing 400 bp homology sequence in the DNA module for homologous recombination. By contrast, in this study, we used a 50 bp homology sequence in the DNA module along with CRISPR/Cas9. Despite this much shorter homology sequence, we achieved duplication of up to 400 kb. We believe that introduction of a DSB mediated by CRISPR/Cas9 enabled at least 400 kb duplication even when a relatively short 50 bp homology sequence was employed. Thus, in the absence of CRISPR/Cas9, a 50 bp homology might be insufficient for successful homologous recombination to occur.

**Table 4:** CRISPR-PCDup can duplicate up to 400kb of chromosomal region

Size of the duplication (Name of region)	gRNA	No. of transformants	Proportion of correct transformants
400 kb (Chr15-L1)	+	25	20% (2/10)
	–	0	–
350 kb (Chr15-L2)	+	40	20% (2/10)
	–	0	–
300 kb (Chr15-L3)	+	733	3% (1/35)
	–	0	–
250 kb (Chr15-L4)	+	114	20% (2/10)
	–	0	–
200 kb (Chr15-L5)	+	120	90% (9/10)
	–	0	–

## Discussion

In this study, we have developed a novel chromosome engineering technology by combining CRISPR/Cas9 system with our previously developed PCDup technology which we called CRISPR-PCDup. Since integration of CRISPR/Cas9 system into PCDup method may increase homologous recombination frequency, we expected that CRISPR-PCDup enables targeting multiple chromosomal regions to be duplicated by a single transformation. Previously, DiCarlo et al. (2013) reported that foreign donor DNA was integrated with nearly 100% frequency at the target site when a DSB is induced by CRISPR/Cas9 in *S. cerevisiae*. Indeed, in this study we found that duplication efficiency was increased approximately 25 to 30 fold when targeting a single site with the help of CRISPR/Cas9 (Table 3). In addition, the proportion of transformants analyzed with the desired karyotype by conventional PCDup was 0%. By contrast, 100% of randomly selected transformants obtained using the CRISPR-PCDup method possessed the anticipated chromosomal changes (Table 3). Although this technology has not yet been tested for other chromosomal regions, we suppose that CRISPR-PCDup may duplicate any chromosomal regions with significantly greater efficiency than conventional PCDup. Besides, a significant increase in the efficiency of a single duplication event is probably the reason for the success of simultaneous double duplications of at least two large chromosomal regions. We believe the enhanced efficiency of this new method arises from the DSBs induced by CRISPR/Cas9 that stimulate an increased rate of homologous recombination.

Next, we attempted simultaneous duplication of two sets of chromosomal regions named as Chr3-1 and Chr8-1 as well as Chr3-1 and Chr14-4 but we did not get simultaneous double duplications in these two cases. Natesuntorn et al. (2015) proposed that duplicating chromosomal regions requires chromosome nondisjunction as one of possible mechanisms. It is likely that incorporation of CRISPR/Cas9 has influence on homologous recombination but not that on chromosome nondisjunction. Therefore, we think that the frequency of chromosome nondisjunction is the same even if we incorporated CRISPR/Cas9 system into our PCDup method. In this case, if the frequency of homologous recombination is not so high in two target sites, we may not get double duplication. By contrast, since sequential duplication needs only one homologous recombination for each transformation followed by possible chromosome nondisjunction, we think that this may be the reason by which we got duplication of Chr3-1 and Chr8-1 as well as Chr3-1 and Chr14-4 regions in a sequential manner but not simultaneously. On the other hand, we got success to duplicate Chr3-2 and Chr15-L5 regions simultaneously when we incorporated CRISPR/Cas9 system. Therefore, we think that possible reason for getting this success of obtaining double duplication simultaneously is that the frequency of homologous recombination became significantly higher by using CRISPR/Cas9 system compared with that of conventional PCDup method. Although it is not so easy to directly estimate the frequency of homologous recombination which is needed to duplicate multiple chromosomal regions simultaneously, we suggest that increased frequency of homologous recombination may contribute to the success of getting simultaneous duplication of Chr3-2 and Chr15-L5 regions.

In this study, we were able to lengthen the regions to be duplicated to 400 kb which is 100 kb larger than the longest duplication (300 kb) by conventional PCDup. In a previous study (Natesuntorn et al. 2015), we proposed that the upper-size limitation of chromosome duplication might be controlled by the frequency of chromosome nondisjunction because the rate of chromosome nondisjunction decreases as the length of the chromosome increases (Hieter et al. 1985). According to this data, larger duplicated chromosomes give rise to decreased rate of chromosome nondisjunction, but we believe that the number of resultant duplicated regions was increased significantly by CRISPR/Cas9 before chromosome nondisjunction occurs. As a result, chance of obtaining a longer duplicated chromosome might be increased and we think that this is the reason why we got viable transformants harboring 400 kb duplicated chromosome.

Simultaneous segmental duplication of multiple chromosomal regions is not reported in any organism. Here, we have demonstrated for the first time that it is possible to simultaneously duplicate two large segments of chromosomal regions (160 kb and 200 kb) using our newly developed CRISPR-PCDup technology. Since segmental aneuploidy are occasionally found in industrial yeast strains displaying robustness (Borneman et al. 2011, Dunn et al. 2012) and we also previously revealed that duplicating several chromosomal regions gives rise to stress resistance against ethanol, high temperature, acetic and sulfuric acid (Natesuntorn et al. 2015), we think that CRISPR-PCDup technology should contribute to investigating combinatorial effect of segmental aneuploidy in an efficient way. Moreover, since duplicated chromosomes act as independent chromosomes comprising extra-copies of many genes, those chromosomes may be suitable for studying the effect of over-expression of many genes on cell physiology. In conclusion, CRISPR-PCDup is a promising tool not only for generating yeast strains that exhibit desired industrial traits but also for studying the fundamentals of genome function.

## Supplementary information

**Additional file 1: Table S1.** Primers used for constructing DNA modules. **Table S2.** Primers used for constructing the gRNA expressing plasmid. **Table S3**. Primers used for Southern blotting. **Table S4.** Positions of duplication points on different chromosomes. **Table S5.** Primers used for colony PCR.

## Data Availability

The datasets generated during and/or analyzed during the current study are available from the corresponding author on reasonable request.
