# Supplementary material for: CRISPR-PCDup: a novel approach for simultaneous segmental chromosomal duplication in Saccharomyces cerevisiae
Source: AMB Express. 2020 Feb 3;10:27. doi: 10.1186/s13568-020-0957-4 (PMC6997328; doi:10.1186/s13568-020-0957-4)
Supplement: Supplementary file 1 — Additional file 1: Table S1. Primers used for constructing DNA modules. Table S2. Primers used for constructing the gRNA expressing plasmid. Table S3. Primers used for Southern blotting. Table S4. Positions of duplication points on different chromosomes. Table S5. Primers used for colony PCR. [file 13568_2020_957_MOESM1_ESM.docx]

| Primer name | Sequence (5’-3’) |
| --- | --- |
| gRNA15-l0-Dup | TACTTATTAACGTACTCAAACAACTACACTTCGTTGTATCTCAGAATGAGGGCCGCCAGCTGAAGCTTCG |
| gRNA15-l1-Dup | TTAGTATTTTGTGTTTTTTACAACAACCTCTCGACTATTGTATACCAGTTGGCCGCCAGCTGAAGCTTCG |
| gRNA15-l2-Dup | GATTTGAACTTTTGTTCTCTCTCTCAACTTTTTCTTTTCTTTGTCCTTGTGGCCGCCAGCTGAAGCTTCG |
| gRNA15-l3-Dup | TATCTGTAGTTTCCTTCCATTACATAACGCATAATATACTATTTCCATAGGGCCGCCAGCTGAAGCTTCG |
| gRNA15-l4-Dup | TACGTTAAAAAAACACATGGTCTTATTTTCCAAAATGCCTATTCCCTATAGGCCGCCAGCTGAAGCTTCG |
| gRNA15-l5-Dup | GACTATAGAAGAAGCGTTCCAGTCAATTCTACTACATCAGTCTTCCGACAGGCCGCCAGCTGAAGCTTCG |
| C3-1 Dup 50 bp | TAATACTGCTGTTGAGGTTTTCTTCTTCAGGGCTGCTCACAACGTGATATGGCCGCCAGCTGAAGCTTCG |
| C3-2 Dup 50 bp | TGTAAGAATATTTGGTATGGCTAAAGTAAGCAAAGCCATATCCCGATCCCGGCCGCCAGCTGAAGCTTCG |
| C5-3 Dup 50 bp | TCTCTTCATAGAGCTCGTCGAAGAGGCAATAGGAACACAACGCCTTACCAGGCCGCCAGCTGAAGCTTCG |
| C8-1 Dup 50 bp | ATTAAAGCGTTAACTCACTCATTATTGTAGCTTATGCGTTTCTCCTCCTCGGCCGCCAGCTGAAGCTTCG |
| C14-4 Dup 50 bp | TTCGCTCAAGTATATTCCGCGTTAATCAACCCACCTGACCCACATTCTAAGGCCGCCAGC TGAAGCTTCG |
| CA Primer | CCCCAACCCCAACCCCAACCCCAACCCCAACCCCAAAGGCCACTAGTGATCTGAT |

**Table S1. Primers used for constructing DNA modules**

**Table S2. Primers used for constructing the gRNA expressing plasmid**

| Primer name | Sequence (5’-3’) |
| --- | --- |
| gRNA15-l0 Fw | TTATAACAAAGCGAACAAAAGTTTTAGAGCTAGAAATAGCAAG |
| gRNA15-l0 Rv | TTTTGTTCGCTTTGTTATAAGATCATTTATCTTTCACTGCGGA |
| gRNA15-l1 Fw | GTAGCATCTATGCAAGAAACGTTTTAGAGCTAGAAATAGCAAG |
| gRNA15-l1 Rv | GTTTCTTGCATAGATGCTACGATCATTTATCTTTCACTGCGGA |
| gRNA15-l2 Fw | TCGTCACAATCTAATCAACAGTTTTAGAGCTAGAAATAGCAAG |
| gRNA15-l2 Rv | TGTTGATTAGATTGTGACGAGATCATTTATCTTTCACTGCGGA |
| gRNA15-l3 Fw | AAAAGATGTAAGATAGACTAGTTTTAGAGCTAGAAATAGCAAG |
| gRNA15-l3 Rv | TAGTCTATCTTACATCTTTTGATCATTTATCTTTCACTGCGGA |
| gRNA15-l4 Fw | ACATATAGGCAAAGATATATGTTTTAGAGCTAGAAATAGCAAG |
| gRNA15-l4 Rv | ATATATCTTTGCCTATATGTGATCATTTATCTTTCACTGCGGA |
| gRNA15-l5 Fw | TAGGATACAATCAGCGATGTGTTTTAGAGCTAGAAATAGCAAG |
| gRNA15-l5 Rv | ACATCGCTGATTGTATCCTAGATCATTTATCTTTCACTGCGGA |
| Ch3 Dup P6-Fw | GGACGTATTCAGCGCAGTTGGTTTTAGAGCTAGAAATAGCAAG |
| Ch3 Dup P6-Rv | CAACTGCGCTGAATACGTCCGATCATTTATCTTTCACTGCGGA |
| Ch3 Dup P1-Fw | AAGGGATCGGAATAAGAGTCGTTTTAGAGCTAGAAATAGCAAG |
| Ch3 Dup P1-Rv | GACTCTTATTCCGATCCCTTGATCATTTATCTTTCACTGCGGA |
| Ch8 Dup P1-Fw | GGATCTTCCACTCCGGTTCGGTTTTAGAGCTAGAAATAGCAAG |
| Ch8 Dup P1-Rv | CGAACCGGAGTGGAAGATCCGATCATTTATCTTTCACTGCGGA |
| Ch14 Dup P1-Fw | GTGTTTAGACTAGGTTTGTC GTTTTAGAGCTAGAAATAGCAAG |
| Ch14 Dup P1-Rv | GACAAACCTAGTCTAAACAC GATCATTTATCTTTCACTGCGGA |

| Primer name | Sequence (5’-3’) |
| --- | --- |
| C3-1-p-f | GCAAGACTCTGGTCTCTTCT |
| C3-1-p-r | ACACCTGAGTGGGTCATCAC |
| C3-2-p-f | CTCTTAGCGGACCGTTTTGG |
| C3-2-p-r | ATCTCTCCGCAGGGGTAAGC |
| C15-5-L-f | CTGCAGCGTACGAAGCTTCAGCTGGCGGCCCCAATTCACAATTTGTCGAT |
| C15-5-L-r | TACAGGTCAATGAAAATGCG |

**Table S3. Primers used for Southern blotting**

**Table S4. Positions of duplication points on different chromosomes**

| Name of region | Chromosome | Nucleotide position | gRNA targeting sequence (5ʹ-3ʹ) |
| --- | --- | --- | --- |
| Chr3-1 | Chr3 | 158050.5 | GGACGTATTCAGCGCAGTTG |
| Chr3-2 | Chr3 | 157533.5 | AAGGGATCGGAATAAGAGTC |
| Chr8-1 | Chr8 | 202256.5 | GGATCTTCCACTCCGGTTCG |
| Chr14-4 | Chr14 | 597353.5 | GTGTTTAGACTAGGTTTGTC |
| Chr15-L0 | Chr15 | 969084.5 | TTATAACAAAGCGAACAAAA |
| Chr15-L1 | Chr15 | 569774.5 | GTAGCATCTATGCAAGAAAC |
| Chr15-L2 | Chr15 | 618913.5 | TCGTCACAATCTAATCAACA |
| Chr15-L3 | Chr15 | 670547.5 | AAAAGATGTAAGATAGACTA |
| Chr15-L4 | Chr15 | 718508.5 | ACATATAGGCAAAGATATAT |
| Chr15-L5 | Chr15 | 767985.5 | TAGGATACAATCAGCGATGT |

| Primer name | Chromosome number (coordinates) | Nucleotide sequence (5’-3’) |
| --- | --- | --- |
| SJP 119 | *CgLEU2* (776-800) Rv | CCCACTAGTTCTCTAACAACGACGA |
| SJP 121 | *CNE1* (211-230) Fw | TCACAGGGTCGATTGCAAGG |
| SJP 242 | *CNE1* (880-861) Rv | CTGGTGGTTCAGTGCCATCT |
| SJP 411 | *CgHIS3* (401-425) Rv | CGCCTCCTTGAACGCTTGGCCCAGC |
| SJP 510 | Chr3-1 (157820-157844) Fw | GCTACATAGCGTTCATTTTT TAGGT |
| SJP 550 | *URA3* check (116545-116569) Rv | GCTTCAAACCGCTAACAATACCTGG |
| SJP 555 | Chr8-1 (201541-201565) Fw | AAAAAATGTGGGATGAAGACTCCCG |
| SJP 668 | Chr14-4 (597970-597994) Rv | ATGGAGAGCACAATCCAGCTTCTTA |

**Table S5. Primers used for colony PCR**
